# Titin modulation and left ventricular remodelling in chronic primary mitral regurgitation

**DOI:** 10.3389/fcvm.2026.1759286

**Published:** 2026-06-03

**Authors:** Lobke L. Pype, Melissa Herwig, Sven L. Van Laer, Dina De Bock, Inez Rodrigus, Bernard P. Paelinck, Hanne M. Boen, Caroline M. Van De Heyning, Nazha Hamdani, Emeline M. Van Craenenbroeck

**Affiliations:** 1 Department of Cardiology, University Hospital Antwerp, Edegem, Belgium; 2 Cardiovascular Diseases, GENCOR Research Group, University of Antwerp, Antwerp, Belgium; 3 Medical Faculty, Institute of Physiology, Department of Cellular and Translational Physiology, Ruhr-University Bochum, Bochum, Germany; 4 Department of Cardiac Surgery, University Hospital Antwerp, Edegem, Belgium; 5 Cardiovascular Surgery, ASTARC Research Group, University of Antwerp, Antwerp, Belgium; 6 Department of Cardiology and Rhythmology, Ruhr-University Bochum, St. Josef-Hospital, Medizinische Klinik II, Bochum, Germany; 7 Department of Physiology, Cardiovascular Research Institute, Maastricht University, Maastricht, Netherlands

**Keywords:** cardiomyopathy, left ventricular remodelling, mitral regurgitation, mitral valve prolapse, titin

## Abstract

**Background:**

Left ventricular (LV) remodelling in mitral valve prolapse (MVP) is usually induced by chronic mitral regurgitation (MR), however it can also be disproportionate to the volume load, generating the hypothesis of MVP cardiomyopathy. Changes in the sarcomeric protein titin could contribute to its underlying pathophysiology.

**Objectives:**

To investigate the role of titin modulation as a mechanism of disproportionate LV remodelling in MVP patients.

**Methods:**

Myocardial biopsies from 16 patients with MVP and severe MR were compared with 6 controls. All patients underwent pre-operative transthoracic echocardiography and cardiac magnetic resonance imaging. Titin modifications were analyzed by gel electrophoresis and western blotting.

**Results:**

Five patients had disproportionate LV remodelling. Compared to controls, the larger N2BA isoform was significantly upregulated in MVP patients, displaying a significantly higher N2BA/N2B isoform ratio (0.589 ± 0.055 vs. 0.479 ± 0.024, *p* < 0.001). The proportion of total N2B-phosphorylated titin was significantly lower in MVP patients with normal vs. disproportionate LV remodelling (0.709 ± 0.142 vs. 0.971 ± 0.084, *p* = 0.008) and controls (1.246 ± 0.147, *p* < 0.001). Furthermore, titin oxidation was significantly higher in MVP patients vs. controls (0.635 ± 0.104 vs. 0.481 ± 0.105, *p* = 0.006). Finally, there was less N2B-titin ubiquitination in MVP compared to controls (0.607 ± 0.138 vs. 0.989 ± 0.102, *p* < 0.001). Univariate linear regression showed that corrected LV end-diastolic volume index was correlated with total N2B-titin phosphorylation (R^2^ = 0.362, *p* = 0.014), suggesting lower passive stiffness in disproportionate LV remodelling.

**Conclusions:**

MVP patients with severe MR demonstrate significant changes in titin isoform ratio, phosphorylation, oxidation and ubiquitination compared to controls. Disproportionate LV remodelling was correlated with increased phosphorylation and showed a trend towards increased N2BA titin, both markers of decreased myocardial stiffness.

## Graphical Abstract

**Figure:**
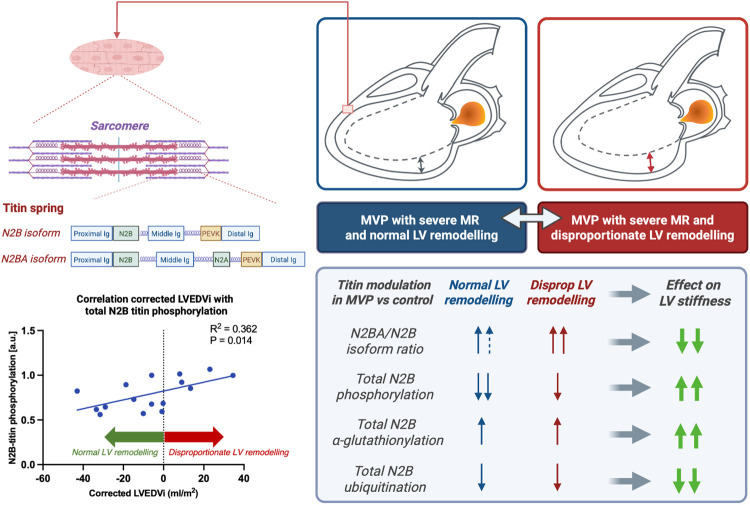
Created in BioRender. Pype L. (2025) https://BioRender.com/0d0vc1r. LV, left ventricular; LVEDVi, left ventricular end-diastolic volume index; MR, mitral regurgitation; MVP, mitral valve prolapse.

## 1 Introduction

Mitral Valve prolapse (MVP) is a common valvular disorder and the most frequent cause of severe primary mitral regurgitation (MR) (1). At one end of the spectrum, Barlow's Disease (BD) occurs in younger patients and is characterized by dilatation of the mitral annulus and the elongation, thickening and prolapse of both leaflets whereas fibroelastic deficiency (FED) occurs in older patients and is characterized by single leaflet or segment prolapse, chordal elongation or rupture, and thickening of the prolapsing leaflet segments (2).

Significant LV remodelling is an important disease feature and is associated with worse prognosis in MVP patients (3, 4). Whereas chronic volume overload due to significant MR has been considered to be the main mechanism of LV remodelling in MVP, other factors are at play (5). Especially in patients with BD, the observation that LV dilatation occurs even in the absence of significant MR, has led to the concept of “disproportionate LV remodelling in MVP” (6–8). Several hypotheses have been put forward to explain this concept, like ventricular arrhythmia-induced cardiomyopathy (9, 10) and/or a concomitant genetic cardiomyopathy (11) but the exact pathophysiological process remains elusive. Understanding and identifying this phenotype is of major importance since correct assessment of underlying risk factors can lead to more optimal, patient tailored, follow-up and therapeutic strategies beyond reduction of MR volume load.

The giant protein titin is known to act as a molecular spring within the sarcomere and therefore has a crucial role in regulating myocardial stiffness (12, 13). In cardiac diseases like dilated or hypertrophic cardiomyopathy the elastic properties of titin can be pathologically altered (12). In human myocardium, there are two titin isoforms present: the smaller and more rigid N2B and larger and more compliant N2BA isoform, usually with an N2BA/N2B distribution of 30/70 in cardiomyocytes (14). In heart failure with severe LV dilatation, upregulation of the more compliant N2BA isoform and thus increased N2BA/N2B ratio has been observed (15–17). Furthermore, an increased N2BA/N2B ratio has been observed in patients with volume overload due to aortic regurgitation, compared to aortic stenosis and controls (18). In contrast, the ratio is known to decrease in settings of severe diastolic dysfunction (12). Apart from isoform switching, myocardial elasticity also depends on other post-translational changes, like phosphorylation, oxidation and ubiquitination (12). Of these pathophysiological processes, phosphorylation processes in titin's I-band extensible regions are known to increase or decrease passive stiffness depending on which domain is affected and which kinase is involved. More specifically, phosphorylation of the N2B domain generally lowers titin-related myocardial passive stiffness (19) whereas phosphorylation of the PEVK domain increases it (20). Both in patients with dilated and hypertrophic cardiomyopathy, reduced N2B-titin phosphorylation has been associated with increased passive stiffness (21, 22). In addition, titin oxidation has been investigated as a mechanism contributing to myocardial stiffness and diastolic dysfunction in animal models of ischemic heart disease and conditions of increased preload and afterload (23–25). More recently, the process of titin ubiquitination or degradation has shown to be altered in hypertrophic cardiomyopathy and in conditions of cardiac volume overload (26, 27).

While alterations in titin related myocardial elasticity have already been observed in cardiomyopathies and aortic valve disease (15, 18, 28), the role of titin in LV remodelling within the context of chronic primary MR remains unknown. Furthermore, whether titin alterations could explain why some patients with MVP present with LV remodelling disproportionate to the degree of MR severity, is another pending question. Therefore, this study was designed to investigate the difference in titin properties from myocardial biopsies in patients with MVP and severe chronic MR, compared to healthy controls. In addition, patients with MVP and disproportionate LV remodelling were identified and compared with normal LV remodelling.

## 2 Methodology

### 2.1 Patient Inclusion

Sixteen patients were prospectively included between August 2021 and July 2024 at a single tertiary center. All patients were diagnosed with severe chronic primary MR (Carpentier II) due to MVP and referred for mitral valve repair/replacement. MVP was defined as an end-systolic displacement of ≥2 mm of (one or both) of the mitral valve leaflets into the left atrium. Subjects were excluded if one of the following criteria were present: history of cardiac surgery, coronary artery disease, significant concomitant left-sided valve disease (≥moderate aortic stenosis/insufficiency and/or ≥ moderate mitral stenosis), syndromic MVP (e.g., Marfan's syndrome), congenital heart disease, known hypertrophic or dilated cardiomyopathy, history of myocarditis, permanent atrial fibrillation, severe kidney dysfunction (eGFR < 30 mL/min), pregnancy and claustrophobia.

Patients were classified as BD or FED according to the intra-operative diagnosis of myxomatous mitral valve (=BD) or overall normal valvular tissue (=FED).

The control group (*n* = 6; male; average age, 43 years) included non-failing donor hearts (LV) with no known history of cardiac disease and with preserved cardiac function prior to organ retrieval. These hearts were not used for transplantation due to technical or logistical reasons rather than cardiac pathology (no history of cardiac disease, a normal ECG and normal ventricular function on echocardiography). The study was approved by the local Research Ethics Committee (University Hospital Antwerp, 21/10/148) and complies with the declaration of Helsinki. All patients provided written informed consent.

### 2.2 Echocardiographic Measurements & calculations

All patients underwent transthoracic echocardiography (TTE) prior to mitral valve surgery (Philips Epiq Ultrasound, Eindhoven, The Netherlands). LV volumes and ejection fraction (LVEF) were calculated using the modified Simpson's method*.* All cardiac volumes were indexed to body surface area (BSA). LV global longitudinal strain (GLS) was assessed using post-processing software package (TomTec, Unterschleißheim, Germany).

Diastolic LV function was assessed according to guidelines (29). Systolic pulmonary artery pressures were calculated from the maximal tricuspid regurgitant jet velocity and an estimate of the right atrial pressure based on inferior caval vein dimension and collapsibility. MR severity was quantified using a multi-integrative approach and graded according to international guidelines (30). All echocardiographic images were analyzed by a single observer.

### 2.3 Cardiac Magnetic resonance measurements & calculations

All patients underwent a cardiac magnetic resonance (CMR) scan less than three months before mitral valve surgery. CMR scans were performed using either a 1.5 T Sola or Aera (Siemens Healthineers, Erlangen, Germany) scanner. CMR protocol included cine-images in all views, through-plane phase-contrast of the ascending aorta to measure forward stroke volume and 10 min after intravenous injection of a gadolinium-based contrast agent, both dark-blood and conventional bright-blood late gadolinium enhancement (LGE) were obtained as described previously (31, 32). T1 mapping was performed and extracellular volume (ECV) was calculated as recommended by international guidelines (33). Ventricular volumes and MR volume were calculated as previously described (31) using a commercially available certified software package CVI42 (Circle Cardiovascular Imaging Inc., Calgary, Canada). In addition, LV end-diastolic volume (LVEDV) was corrected for the respective volume load (= MR volume), and body surface area (BSA) and then compared to the age- and sex-specific upper limit of normal (LVEDV_ULN_) (34). Disproportionate LV remodelling was defined as LV dilatation beyond what would be expected based on the underlying MR volume load and the respective age and sex of the patient. This was calculated using the following formula: (LVEDV—MRvol)/BSA—LVEDVi_ULN_ > 0.

### 2.4 Biopsy Procedure

Myocardial biopsies from MVP group were taken during the surgical mitral valve procedure. Full thickness specimens were taken from the free LV wall using biopsy forceps. Biopsy samples were snap-frozen in liquid nitrogen and stored in a −80 °C freezer.

Donor control samples (LV myocardium of hearts) were collected in cardioplegic solution, stored in liquid nitrogen until use, and processed using standardized and identical protocols across groups to reduce pre-analytical variability.

### 2.5 Titin Gel electrophoresis: titin N2B and N2BA isoforms

Titin isoforms were separated as previously described (21, 35)*.* Briefly, tissue samples were solubilized in a modified Laemmli buffer (0.05 M Tris-HCl pH 6.8, 8 M urea, 2 M thiourea, 3% SDS (*w*/*v*), 0.03% ServaBlue (*w*/*v*), 10% (*v*/*v*) glycerol, 75 mM DTT). Samples were heated for 3 min at 96 °C and centrifuged (14.000 rpm, 3 min). Then, samples (20 *μ*g; equal concentration checked by spectroscopic methods using PierceTM 660 nm protein assay [Thermo Fisher Scientific, Waltham, MA, USA)] were separated by agarose-strengthened 2% sodium dodecyl sulfate polyacrylamide gel electrophoresis (SDS-PAGE). Gels were run at 2–4 mA constant current for 16 h. To determine titin expression, titin gels were stained for one hour with Serva Blue R (SERVA Electrophoresis GmBH, Heidelberg, Germany) Staining was visualized using ChemiDoc Imaging system (BioRad, Munich, Germany) and signals were analysed using Multi Gauge V3.2 (FUJIFILM Corp, Minato, Tokyo, Japan). Titin-isoform composition was expressed assuming that the isoforms N2BA and N2B together make up 100% of intact titin. Each sample was analyzed in technical duplicate, and the resulting densitometric values were averaged prior to statistical analysis. Only titin gels within the linear dynamic range of detection were used for quantification.

### 2.6 Titin Immunoblots: titin phosphorylation, oxidation and ubiquitination

Western blots were performed using custom-made, affinity-purified antibodies to evaluate titin phosphorylation, oxidation and ubiquitination as previously described (26). After SDS-PAGE, gels were blotted onto polyvinylidene difluoride membranes (PVDF; Immobilon-*P* 0.45 μm; Merck Millipore, Burlington, MA, USA). Transfer and staining uniformity were visually inspected and quantitatively verified across all lanes. Blots were blocked with 5% bovine serum albumin in Tris-buffered saline with Tween (TBST) for 1 h at RT and subsequently incubated with the respective antibodies. The total N2B phosphorylation was assessed using anti–phospho-serine/threonine antibodies (ECM Biosciences LLC; PP2551; 1:500 dilution). To assess titin oxidation (*α*-glutathionylation) anti-GSH antibodies (ab19534, Abcam, 1:500 dilution) were used. Finally, anti-ubiquitin antibodies (43,124, Cell Signaling Technology, 1:1,000 dilution) were used to assess titin ubiquitination. After washing with TBST, primary antibodies were detected with horse radish peroxidase-conjugated secondary goat anti-rabbit (dilution 1:10,000; OriGene Technologies GmbH) or anti-mouse antibodies (dilution 1:10,000; Jackson ImmunoResearch Europe Ltd.) and enhanced chemiluminescence using the Clarity Western ECL Substrate (BioRad, Munich, Germany). Chemiluminescence signals were normalized to signals obtained from Coomassie-stained PVDF membranes referring to the entire protein amount transferred. Stained protein band were quantified via densitometry using Multi Gauge V3.2 software. The obtained density values are expressed in arbitrary units [a.u.]. Each sample was analyzed in technical duplicate, and the resulting densitometric values were averaged prior to statistical analysis. Only titin blots within the linear dynamic range of detection were used for quantification.

### 2.7 Statistics

Continuous Variables are expressed as mean ± standard deviation for normally distributed variables or median with interquartile range (IQR) for parameters with a non-normal distribution. Titin data are expressed as mean ± standard error of mean. Categorical variables are expressed as numbers and percentages and were compared using a Chi-square test or Fisher's exact test. Comparisons between two groups (disproportionate vs. normal LV remodelling and control vs. MVP samples) were performed with a Student's *t*-test or nonparametric alternative (Mann–Whitney *U* test). Univariable linear regression analyses were conducted to identify the determinants of LVEDVi. A *p*-value of ≤ 0.05 was considered statistically significant. Statistical analyses were performed using SPSS version 28.0 (SPSS Inc., Chicago, IL, USA) and GraphPad Prism 5 (GraphPad Software, Boston, Massachusetts, USA).

## 3 Results

### 3.1 Clinical Data and cardiac imaging

Clinical characteristics and cardiac imaging results are shown in Table 1. A total of 16 MVP patients were included (mean age of 62 ± 10 years old and 13 males), compared to six control patients (mean age 58 ± 6 years old, 2 males). Fibroelastic deficiency phenotype was present in 10 patients (62.5%) and Barlow's Disease in the other 6 patients (37.5%). MVP patients presented with increased LV end-systolic and end-diastolic volumes as measured with CMR (compared to reference values, see Table 1), as well as increased left atrial volumes. Echocardiographic assessment showed varying degrees of diastolic dysfunction in MVP patients: 25% with impaired relaxation (grade 1), 43.2% with pseudonormal pattern (grade 2) and 31.1% with restrictive filling (grade 3). Severe MR was present based on calculated MR volume and regurgitant fraction using CMR. Myocardial LGE was present in 62.5% and mean ECV values were high normal.

**Table 1:** Baseline clinical & imaging characteristics.

	All MVP patients (*n* = 16)	Normal LV remodelling (*n* = 11)	Disproportionate LV remodelling (*n* = 5)	Reference values	*P* value *
Age (y)	62 ± 10	63 ± 10	61 ± 9		0.691
Male sex, *n* (%)	13 (81.2)	9 (81.2)	4 (80.0)		NS
BSA (m^2^)	1.98 ± 0.24	1.94 ± 0.26	2.08 ± 0.20		0.313
MVP subtype					
Barlow's Disease, *n* (%)	6 (37.5)	3 (27.3)	3 (60.0)		0.299
Fibroelastic deficiency, *n* (%)	10 (62.5)	8 (72.7)	2 (40.0)		
Medication					
Beta-blocker, *n* (%)	5 (31.1)	4 (36.4)	1 (20.0)		NS
ACE-inhibitor or ARB, *n* (%)	8 (50.0)	5 (45.5)	3 (60.0)		NS
Mineralocorticoid-receptor antagonist, *n* (%)	2 (12.5)	2 (18.2)	0 (0.0)		NS
Diuretics, *n* (%)	3 (18.8)	3 (27.3)	0 (0.0)		NS
Echocardiography					
LAVI (mL/m^2^)	58.3 ± 18.4	57.6 ± 19.6	60.3 ± 17.5		0.816
LVEF (%)	61.2 ± 5.2	62.0 ± 4.8	59.1 ± 6.7		0.360
LV GLS (%)	−20.3 ± 2.9	−20.6 ± 3.2	−19.4 ± 2.3		0.286
Diastolic dysfunction, *n* (%)					
Grade 1	4 (25.0)	2 (18.2)	2 (40.0)		0.836
Grade 2	7 (43.8)	6 (54.5)	1 (20.0)		
Grade 3	5 (31.3)	3 (27.3)	2 (40.0)		
E (cm/s)	115.4 ± 33.8	111.9 ± 24.7	116.4 ± 52.1		0.695
E/A	2.0 ± 0.8	1.9± 0.9	2.0 ± 0.8		0.433
Septal e' (cm/s)	7.9 ± 1.8	8.0 ± 1.9	8.8 ± 2.7		NS
Lateral e' (cm/s)	10.6 ± 4.0	10.2 ± 4.0	10.4 ± 4.7		0.472
Mean E/e'	13.6 ± 4.2	13.5 ± 4.1	12.7 ± 5.0		0.794
IVRT (ms)	73.0 ± 16.0	73.5 ± 16.4	72.0 ± 16.9		0.873
sPAP (mmHg)	34.1 ± 13.4	34.0 ± 12.6	34.2 ± 18.0		0.975
CMR					
LVEDVi (mL/m^2^)	121.6 ± 27.9	109.2 ± 14.7	148.8 ± 32.0	♂77 ± 15^#^ ♀69 ± 12^#^	0.004
LVESVi (mL/m^2^)	49.2 ± 14.1	43.1 ± 8.9	62.4 ± 15.1	♂29 ± 9^#^ ♀24 ± 7^#^	0.006
LVEF (%)	60.5 ± 4.4	60.7 ± 5.0	59.9 ± 3.1	♂63 ± 3^#^ ♀66 ± 7^#^	0.743
LV mass index (g/m^2^)	65.0 ± 9.4	62.9 ± 5.6	69.6 ± 14.6	♂56 ± 10^#^ ♀45 ± 7^#^	0.201
MR Reg vol (mL)	65.7 ± 31.4	59.0 ± 17.9	80.4 ± 50.1		0.401
MR RF (%)	44.4 ± 12.0	45.8 ± 12.0	41.4 ± 13.0		0.515
LGE present, *n* (%)	10 (62.5)	7 (63.6)	3 (60.0)		NS
Native T1 time mean (ms)	1,019.2 ± 23.2	1,017.1 ± 24.2	1,023.6 ± 22.6		0.625
ECV (%)	27.4 ± 1.9	27.5 ± 2.2	27.4 ± 0.9		0.934

* *P*-value: normal vs. disproportionate LV remodelling. Between group comparisons were made using Chi square or Fischer's exact test for categorical variables and Student's *T*-test for continuous variables.

# Reference values for males and females derived from Kawel Boehm et al. (40).

ACE, angiotensin converting enzyme; ARB, angiotensin receptor antagonist; BSA, body surface area; ECV, extracellular volume; GLS, global longitudinal strain; LGE, late gadolinium enhancement; IVRT, isovolumic relaxation time; LAVI, left atrial volume index; LVEDVi, left ventricular end-diastolic volume index; LVESVi, left ventricular end-systolic volume index; LVEF, left ventricular ejection fraction; MR Reg Vol, mitral regurgitant volume; MR RF, mitral regurgitant fraction; SPAP, systolic pulmonary artery pressure.

Disproportionate LV remodelling was identified in 5 patients (31%) (Table 1). These patients had significantly higher LVEDVi and LVESVi compared to patients with normal LV remodelling, but similar LVEF. Furthermore, left atrial volumes were similar in both groups.

In addition, MR regurgitant volume (calculated using CMR) was not significantly larger in patients with disproportionate vs. normal LV remodelling and regurgitant fraction was similar.

The degree of diastolic dysfunction and corresponding parameters were similar across both groups (see Table 1). The presence of myocardial LGE, native T1 time and ECV did not show a significant difference in normal compared to disproportionate LV remodelling.

### 3.2 Titin Isoform ratio and expression

**Figure 1:**
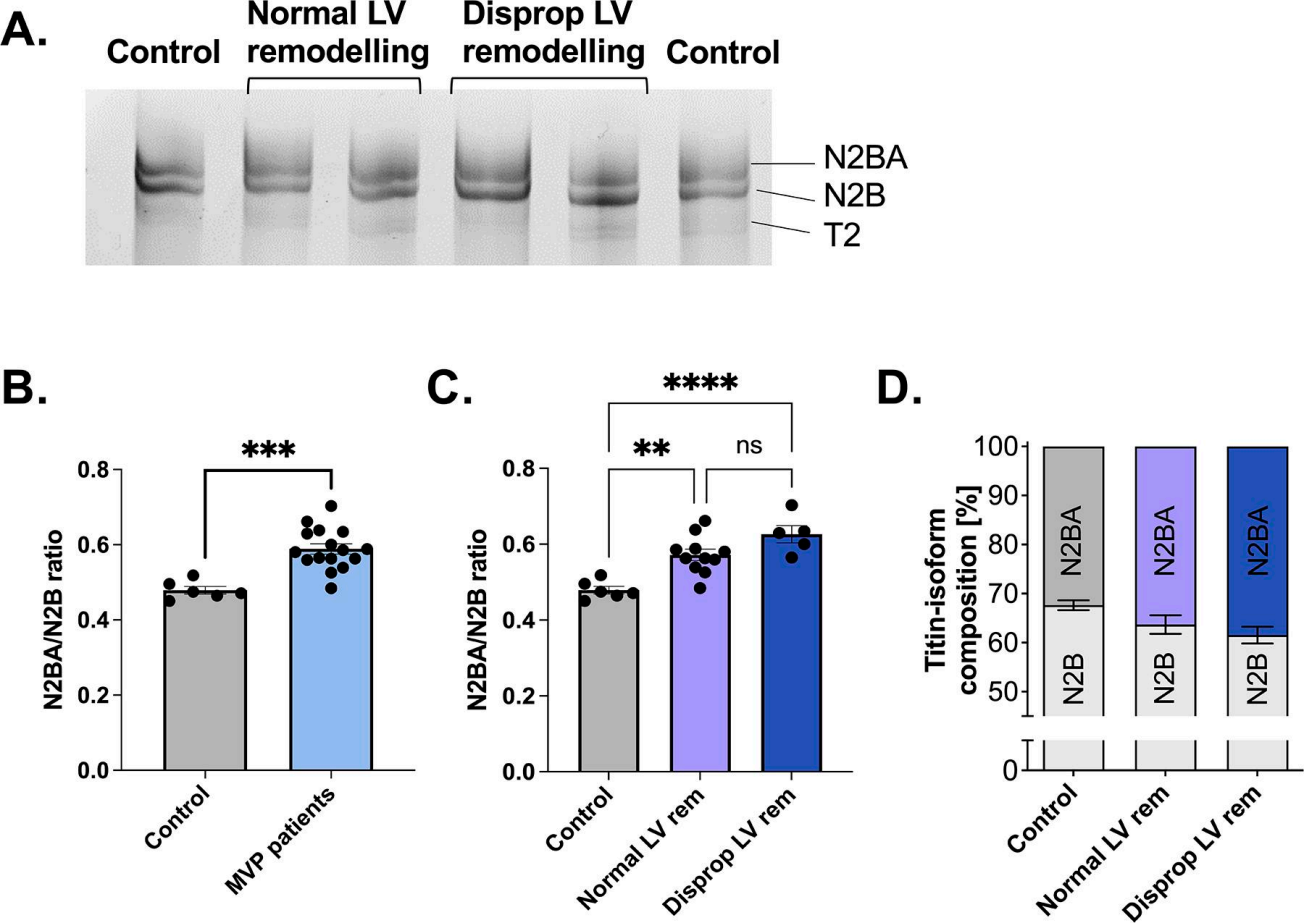
Titin N2BA/N2B isoform ratio in human myocardial tissue. Panel **(A)**: gel electrophoresis showing N2BA, N2B and T2 titin isoform bands. Panel **(B)**: N2BA/N2B ratio in controls vs. MVP. Panel **(C)**: N2BA/N2B ratio in controls vs. normal LV remodelling and disproportionate LV remodelling. Panel **(D)**: titin isoform ratio with proportion of N2B and N2BA in controls vs. normal LV remodelling and disproportionate LV remodelling. Original blots/gels are presented in Supplementary Figure S1. Data are shown as mean ± SEM; ns: *p* > 0.05; *: *p* ≤ 0.05; **: *p* ≤ 0.01; ***: *p* ≤ 0.001; ****: *p* ≤ 0.0001. Between group comparisons were performed using non-parametric tests due to small sample-size (Mann–Whitney *U* Test). LV, left ventricular; MVP, mitral valve prolapse; T2, titin degradation product.

Table 2 and Figure 1 show titin N2BA and N2B titin expression and ratio in patients and controls. In MVP patients, the proportion of the N2BA isoform was increased and the N2B isoform was decreased, compared to controls (Figure 1 panel B). Accordingly, the ratio of N2BA/N2B was 0.589 ± 0.055 in MVP patients vs. 0.479 ± 0.024 in controls (*p* < 0.001). Patients with disproportionate LV remodelling did not have a significantly higher proportion of N2BA compared to normal LV remodelling and N2BA/N2B isoform ratio was not significantly higher (0.627 ± 0.051 vs. 0.572 ± 0.049, *p* = 0.062), as is shown in Figure 1 panel C,D.

**Table 2:** Titin isoform ratio, phosphorylation, glutathionylation and ubiquitination.

	Controls (*n* = 6)	All MVP patients (*n* = 16)	Normal LV remodelling (*n* = 11)	Disproportionate LV remodelling (*n* = 5)	*P* value°	*P* value*
N2BA (%)	32.38 ± 1.09	36.99 ± 2.16	36.32 ± 1.99	38.46 ± 1.91	<0.001	0.062
N2B (%)	67.63 ± 2.16	63.01 ± 2.16	63.68 ± 1.99	61.54 ± 1.91	<0.001	0.062
Ratio N2BA/N2B	0.479 ± 0.024	0.589 ± 0.055	0.572 ± 0.049	0.627 ± 0.051	<0.001	0.062
Total N2B-titin phosphorylation [a.u.]	1.246 ± 0.147	0.915 ± 0.265	0.709 ± 0.142	0.971 ± 0.084	<0.001	0.008
Total N2B-titin glutathionylation [a.u.]	0.481 ± 0.105	0.635 ± 0.104	0.605 ± 0.097	0.703 ± 0.096	0.006	0.100
Total N2B-titin ubiquitination [a.u.]	0.989 ± 0.102	0.607 ± 0.138	0.619 ± 0.141	0.579 ± 0.144	<0.001	0.462

° All MVP patients vs. controls. All non-parametric tests, small sample-size (Mann–Whitney *U* Test to compare 2 groups).

* *P*-value: normal vs. disproportionate LV remodelling. All non-parametric tests. (Mann–Whitney *U* Test to compare 2 groups) Immunoblot density values are expressed in arbitrary units [a.u.].

### 3.3 N2B-titin Phosphorylation

**Figure 2:**
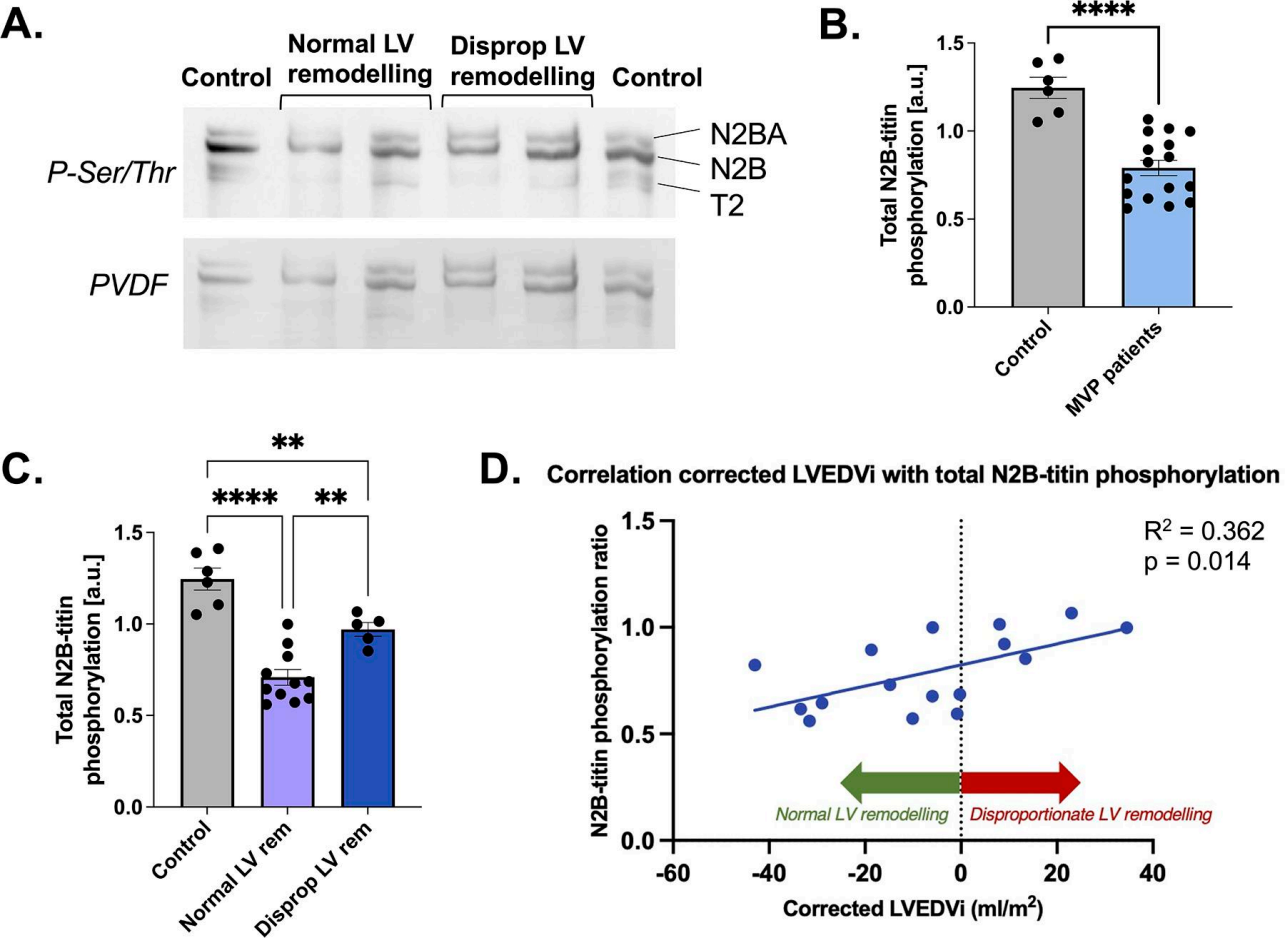
Total N2B-titin phosphorylation in human myocardial tissue. Panel **(A)**: immunoblot using anti–phospho-serine/threonine antibodies and PVDF stain as control. Signals of total phosphorylated N2B-titin (on immunoblot) were normalized to signals obtained from PVDF stain (to compensate for total amount of protein transferred). Panel **(B)**: total N2B-titin phosphorylation in controls vs. MVP (relative to PVDF stain). Panel **(C)**: total N2B-titin phosphorylation in controls vs. normal LV remodelling and disproportionate LV remodelling (relative to PVDF stain). Panel **(D)**: correlation of total N2B-titin phosphorylation (relative to PVDF stain) with corrected LV end-diastolic volume (for mitral regurgitant volume and upper limit of normal) in MVP patients, R^2^ = 0.362, *p* = 0.014. Original blots/gels are presented in Supplementary Figure S2. Data are shown as mean ± SEM; ns: *p* > 0.05; *: *p* ≤ 0.05; **: *p* ≤ 0.01; ***: *p* ≤ 0.001; ****: *p* ≤ 0.0001. Between group comparisons [Panel **(B)** and **(C)**] were performed using non-parametric tests due to small sample-size (Mann–Whitney *U* Test). LV, left ventricular; LVEDVi, left ventricular end-diastolic volume index; MVP, mitral valve prolapse; PVDF, polyvinylidene difluoride; T2, titin degradation product.

Figure 2 shows total N2B-titin phosphorylation in MVP patients vs. controls. Total N2B-titin phosphorylation was significantly decreased by ≈26% in patients with MVP and severe MR, compared to normal controls (0.915 ± 0.265 vs. 1.246 ± 0.147, *p* < 0.001). (Table 2 and Figure 2 panel B) Interestingly, N2B-titin was less phosphorylated in patients with MVP and normal LV remodelling compared to disproportionate LV remodelling (0.709 ± 0.142 vs. 0.971 ± 0.084, *p* = 0.008). (Figure 2 panel C).

### 3.4 N2B-titin *α*-glutathionylation

**Figure 3:**
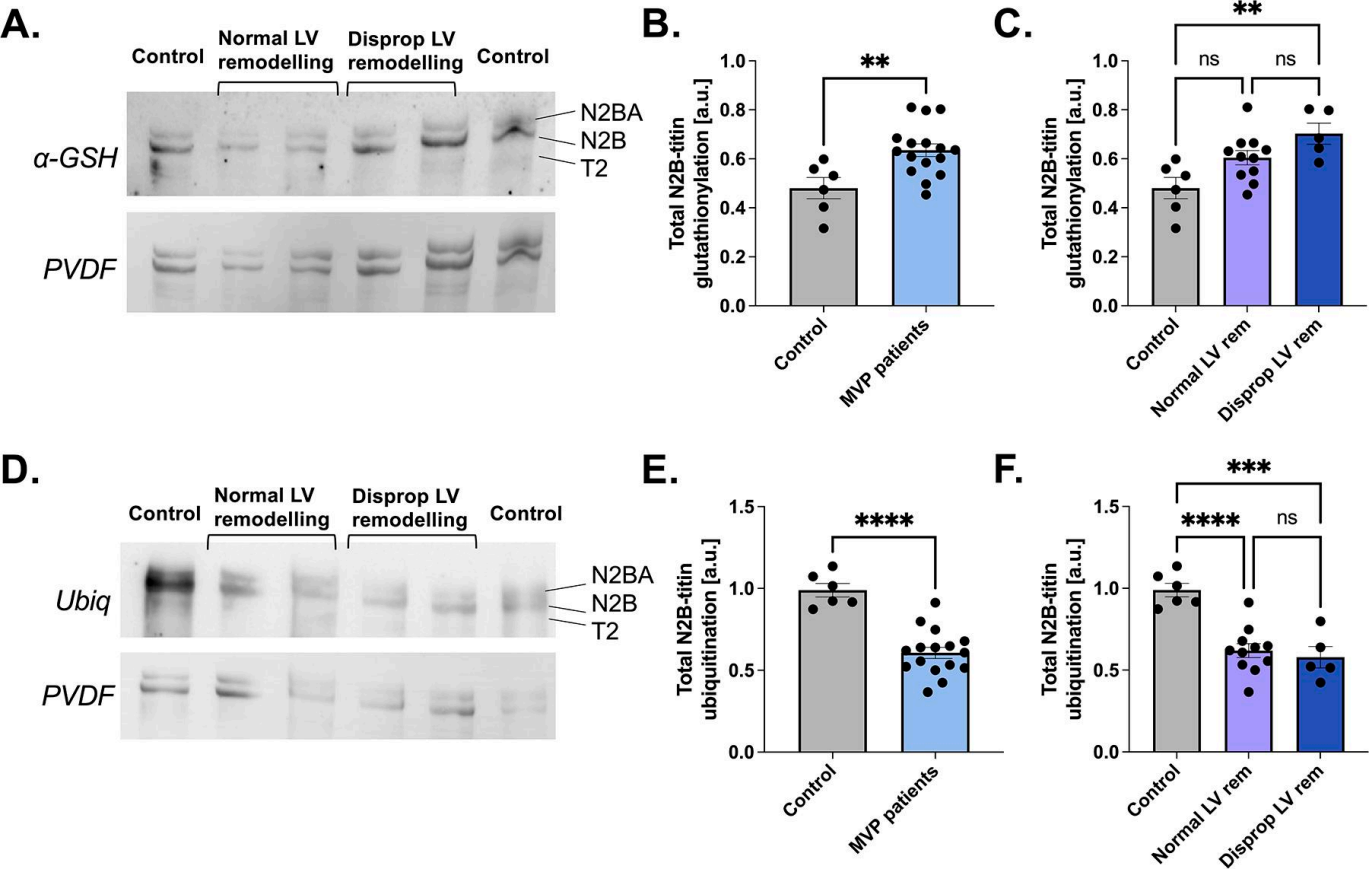
Total N2B-titin glutathionylation and ubiquitination in human myocardial tissue. Panel **(A)**: immunoblot using anti-GSH antibodies and PVDF stain as control. Signals of total glutathionylated N2B-titin (on immunoblot) were normalized to signals obtained from PVDF stain (to compensate for total amount of protein transferred). Panel **(B)**: total N2B-titin glutathionylation in controls vs. MVP (relative to PVDF stain). Panel **(C)**: total N2B-titin glutathionylation in controls vs. normal LV remodelling and disproportionate LV remodelling (relative to PVDF stain). Panel **(D)**: immunoblot using anti-ubiquitin antibodies and PVDF stain as control. Signals of total ubiquinated N2B-titin (on immunoblot) were normalized to signals obtained from PVDF stain (to compensate for total amount of protein transferred). Panel **(E)**: total N2B-titin ubiquitination in controls vs. MVP. Panel **(F)**: relative total N2B-titin ubiquitination in controls vs. normal LV remodelling and disproportionate LV remodelling (relative to PVDF stain). Original blots/gels are presented in Supplementary Figure S3. NS *p* > 0.05; * *p* ≤ 0.05; ** *p* ≤ 0.01; *** *p* ≤ 0.001; **** *p* ≤ 0.0001. Between group comparisons were performed using non-parametric tests due to small sample-size (Mann–Whitney *U* Test). LV, left ventricular; MVP, mitral valve prolapse; PVDF, polyvinylidene difluoride; T2, titin degradation product.

Titin oxidation or *α*-glutathionylation was increased significantly in MVP patients compared to controls (0.635 ± 0.104 vs. 0.481 ± 0.105, *p* = 0.006) (Table 2 and Figure 3 panel A–C). In addition, patients with disproportionate remodelling had higher levels of glutathionylated titin compared to controls (0.703 ± 0.096 vs. 0.481 ± 0.105, *p* ≤ 0.01).

### 3.5 N2B-titin Ubiquitination

The titin degradation process can be assessed by quantifying the proportion of ubiquitinated titin. This was performed using an anti-ubiquitin antibody as previously mentioned. (Figure 3 panel D) We observed less N2B-titin ubiquitination in MVP patients compared to controls (0.607 ± 0.138 vs. 0.989 ± 0.102, *p* < 0.001). (Table 2 and Figure 3 panel E) This difference was already present in patients with normal LV remodelling, and persisted in the disproportionate LV remodelling phenotype. There was no significant difference between these two groups (0.619 ± 0.141 vs. 0.579 ± 0.144, *p* = 0.462). (Figure 3 panel F) In Supplementary Table S1 is shown that presence of focal fibrosis (measured as LGE on CMR) was negatively correlated with N2B-titin ubiquitination (Spearman rho −0.616, *p* = 0.011). There was no significant correlation with diffuse fibrosis (measured as ECV on CMR).

### 3.6 Univariate Linear regression analysis for LV remodelling

Table 3 shows univariate linear regression analysis of the association between titin parameters and LVEDVi. Total N2B-phosphorylation was significantly correlated with LVEDVi (B 96.46, 95% CI 24.46–168.45, *p* = 0.014). The N2BA/N2B isoform ration, titin oxidation and ubiquitination did not show a significant correlation with LV dilatation, even after correction for MR volume and age. Other clinical or imaging parameters like age, gender, diastolic dysfunction or fibrosis did not show a significant correlation either at univariable linear regression analysis. Only MR volume significantly predicted LVEDVi (R^2^ = 0.607, *p* < 0.001). Furthermore, corrected LVEDVi (for MR volume and age- and sex-related upper limit of normal) was also correlated with total N2B-titin phosphorylation at univariate linear regression (R^2^ = 0.362, *p* = 0.014), as is shown in Figure 2 panel D.

**Table 3:** Univariable and multivariable regression analysis for LVEDVi.

	Univariable linear regression		Multivariable linear regression
	B (95% confidence interval)	*P* value	*P* value*
Age (years)	−1.195 (−2.668–0.277)	0.104	
Male gender	19.893 (−18.104–57.890)	0.280	
MR volume (mL)	0.691 (0.372–1.010)	<0.001	
N2BA/N2B ratio	106.43 (−179.25–392.11)	0.438	0.686
Total N2B-titin phosphorylation [a.u.]	96.46 (24.46–168.45)	0.014	0.006
Total N2B-titin glutathionylation [a.u.]	47.00 (−103.89–197.89)	0.515	0.388
Total N2B-titin ubiquitination [a.u.]	29.34 (−85.20–143.88)	0.591	0.500

Immunoblot density values are expressed in arbitrary units [a.u.].

* *P* value after correction for MR volume and age at multivariable linear regression analysis.

## 4 Discussion

For The first time, titin modifications in LV myocardium in patients with LV remodelling due to severe MVP-induced MR were investigated. The main results include that patients with MVP and severe MR present with (1) upregulation of the larger and more compliant N2BA titin isoform and higher N2BA/N2B isoform ratio, (2) reduced total N2B-titin phosphorylation, (3) increased total N2B-titin glutathionylation, and (4) decreased total N2B-titin ubiquitination compared to healthy controls. In addition, patients with disproportionate LV remodelling showed a trend towards upregulation of the N2BA/N2B isoform ratio (*p* = 0.062) and a significant increase in total N2B-titin phosphorylation (*p* = 0.008), both associated with lower passive stiffness, compared to patients with normal LV remodelling.

While LV remodelling in the presence of chronic primary MR originates from the longstanding volume overload, LV dilatation in MVP has also been observed in patients without significant MR, especially in patients with classic MVP or Barlow's Disease (6–8). From these findings the hypothesis was generated that an underlying cardiomyopathy could potentially explain this phenomenon of LV remodelling disproportionate to the degree of MR (5). Therefore, we studied 11 patients with normal LV remodelling and 5 patients with disproportionate LV remodelling (corrected for MR volume and age- and sex-related upper limit of normal) to evaluate potential differences in the sarcomeric protein titin as a pathophysiological mechanism.

Our patient cohort presented with significant LV remodelling in the presence of severe MR, including LV dilatation (mean LVEDVi 121.6 ± 27.9 mL/m^2^) and generally preserved LV systolic function (mean LVEF 60.5 ± 4.4%). Furthermore, echocardiographic assessment showed clinically impactful diastolic dysfunction with mean E/e' of 13.6 ± 4.2 in MVP patients*.*

Compared to healthy controls, we observed several different cardiac titin modifications in MVP patients. When looking at titin isoform switching as regulating mechanism of myocardial elasticity, we found a significant upregulation of the larger, more compliant N2BA isoform and subsequently an increased N2BA/N2B ratio in MVP patients compared to controls (0.589 ± 0.055 vs. 0.479 ± 0.024, *p* < 0.001), indicating reduced myocardial stiffness and increased LV compliance. Interestingly, the N2BA/N2B isoform ratio that we found in our cohort of MVP patients with severe MVP, was lower compared to two cohorts of dilated cardiomyopathy (DCM) patients with end-stage heart failure where they found isoform ratios of 0.670 (16) up to nearly 1 (15). Importantly, these patients presented with a different, more progressed phenotype with LV dilatation and severe LV systolic dysfunction (LVEF < 30%). In addition, they found that the isoform ratio was inversely correlated with degree of diastolic dysfunction with higher N2BA/N2B ratio in impaired relaxation (grade 1) compared to restrictive filling pattern (grade 3) (15). Since our patients presented with varying degrees of diastolic dysfunction of grade 1 or more, this potentially attenuated the increase in N2BA isoform that we would have expected in DCM. Nevertheless, our results were similar compared to a cohort of patients with chronic volume overload due to aortic regurgitation that presented with mean N2BA/N2B ratio of 0.510 (18).

Interestingly, the proportion of the more compliant N2BA isoform was higher in MVP patients with normal LV remodelling (N2BA 36.3%) compared to healthy controls (N2BA 32.4%), and had a nonsignificant trend (*p* = 0.062) to be further elevated in patients with disproportionate LV remodelling (N2BA 38.5%), suggesting that N2BA upregulation could be a continuous process in progressive LV dilatation. Alternative titin splicing into both N2BA and N2B isoforms could act as an adaptive mechanism to ongoing volume overload allowing for larger LV end-diastolic volumes and increased cardiac output while maintaining the same filling pressures (Frank-Starling mechanism).

Besides isoform switching, post-translational titin phosphorylation can be involved in regulating myocardial compliance. Depending on the specific phospho-site, N2B or PEVK domain, the effect of phosphorylation can either be increased or decreased stiffness. As such, protein kinase A and protein kinase G are known to reduce passive stiffness by phosphorylating the N2B-titin domain and heart failure with preserved ejection fraction has shown to be associated with hypophosphorylated N2B-titin (19, 21). In contrast, phosphorylation of the PEVK domain by protein kinase C*α* increases myocardial passive tension (36). This study evaluated the total N2B-titin phosphorylation, which was significantly decreased in MVP patients vs. healthy controls. These results could be compatible with increased diastolic dysfunction and stiffness, as suggested by the echocardiographic measurements. However, we did not investigate different titin phospho-sites or activation by specific protein kinases, nor did we perform any functional testing of myocardial passive stiffness. Therefore, the correlation between N2B-titin phosphorylation and passive stiffness in this cohort should be regarded as merely hypothesis generating.

More specifically, hypophosphorylation of total N2B-titin was already observed in patients with normal LV remodelling, which could potentially be due to downregulation of all phosphorylation pathways, including all protein kinases. However this can only be hypothesized since our study did not investigate any specific phospho-sites in the N2B domain or the effect of specific protein kinases. In contrast, total N2B-phosphorylation was again upregulated in patients with disproportionate LV remodelling, compared to the less severe “normal” LV remodelling phenotype. This could indicate that after the initial process of increasing myocardial stiffness in the presence of diastolic dysfunction, a further reduction in passive stiffness occurs when LV remodelling becomes more disproportionate to the underlying volume load. Given the lack of functional data regarding myocardial passive stiffness in this study, these are only hypotheses on the potential underlying mechanism and should be investigated further. The same pattern was also observed in end-stage DCM patients in whom total N2B-phosphorylation was unchanged compared to controls, in contrast to modest hypophosphorylation in hypertrophic cardiomyopathy patients (36). We hypothesize that the upregulation of other kinases could explain this in the more severe phenotype, but again this was not investigated in the present study. In addition, univariate linear regression analysis showed that corrected LVEDVi (for MR volume and age- and sex-related upper limit of normal) was correlated with total N2B-titin phosphorylation (R^2^ = 0.362, *p* = 0.014), indicating that more disproportionate LV dilatation could be associated with increased N2B-titin phosphorylation and therefore lower passive stiffness.

Furthermore, the process of titin *α*-glutathionylation is known to enhance titin elasticity by reducing the folding of immunoglobulin domains in titin's I-band (23, 24). Increased oxidative stress with s-glutathionylation of titin has been observed in animal studies of adaptive LV remodelling after myocardial infarction, in the presence of increased preload and in human failing hearts (24, 25, 37). Our study showed significantly higher titin *α*-glutathionylation in MVP patients with severe MR vs. controls (0.635 ± 0.104 vs. 0.481 ± 0.105, *p* = 0.006), which could be an indirect marker of higher levels of oxidative stress in the cardiomyocytes. Furthermore, there was a trend towards higher levels of oxidated titin in patients with disproportionate LV remodelling compared to normal LV remodelling (0.703 ± 0.096 vs. 0.605 ± 0.097, *p* = 0.100). However, this was not statistically significant and therefore can only generate a potential hypothesis that increased oxidative stress in the cardiomyocyte occurs in more disproportionate LV remodelling.

Next, ubiquitination is a protein quality control process that initiates degradation of misfolded proteins, like titin, and upregulation of this pathway can occur as response to volume overload in order to protect the cardiomyocyte from proteotoxicity (26). In the present study we observed a significant decrease in ubiquitinated titin in MVP patients compared to controls (0.607 ± 0.138 vs. 0.989 ± 0.102, *p* < 0.001), suggestive of a pathological state in which protein quality control is reduced.

Moreover, MVP patients with normal LV remodelling already showed a significant decrease in ubiquitinated titin compared to controls (0.619 ± 0.141 vs. 0.989 ± 0.102, *p* < 0.001). These changes persisted in patients with the disproportionate LV remodelling phenotype and the effect was similar compared to patients with normal LV remodelling (0.579 ± 0.144 vs. 0.619 ± 0.141, *p* = 0.462). These findings indicate that patients with severe primary MR and associated LV dilatation as expected for the underlying volume load, could already present with significant changes at the cardiomyocyte level promoting cardiotoxicity. Moreover, the presence of focal fibrosis was strongly correlated with reduced N2B-titin ubiquitination (Supplementary Table S1, rho = 0.616, *p* = 0.011), promoting an association between titin ubiquitination and myocardial fibrosis.

Whether the observed alterations in cardiac titin are completely reversible after elimination of the volume load by mitral valve surgery still remains elusive. While in most patients favorable post-operative LV reverse remodelling can be observed, normalization of LV volumes and systolic function doesn't always occur (38, 39). Therefore, it can be hypothesized that in some cases the LV remodelling process and associated cardiomyocyte toxicity has progressed too far prior to the intervention and some of the damage has become irreversible. Whether this could be due to a genetic predisposition or other factors that impact pre-operative LV remodelling has not yet been investigated. Potentially mechanical elimination of the volume load alone is not sufficient to reverse the process of LV remodelling in all patients, but additional medical treatment targeting the underlying molecular mechanisms could be what's needed for recovery of the cardiomyocytes. At present, animal studies have already shown that treatment with anti-oxidant glutathione can reverse myocardial stiffness (26), and therefore pathways involving oxidative stress and protein quality control could be a therapeutic target for novel drug development in the future.

This study highlights the changes in titin modulation underlying LV remodelling in patients with severe primary MR. With increasing disproportionate LV remodelling, we observed a trend towards upregulation of the N2BA titin isoform and more N2B-titin phosphorylation, both of which could be associated with increased myocardial elasticity, although this needs to be assessed further with appropriate functional studies. Whether these changes occur as a consequence of significant LV dilatation or rather predispose patients to more LV dilatation independent of MR volume load, cannot be concluded from our results. Although the concept of disproportionate LV remodelling is not yet fully understood, enhancing the mechanistic insights in this process will increase our knowledge and ability to create a patient-tailored approach on the treatment of the underlying valvular and associated myocardial disease.

Limitations of the present study include the small sample size, not allowing for a more in-depth evaluation of all possible (clinical) factors impacting LV remodelling in MVP patients besides titin modulation. Therefore, our findings should be validated in future studies with larger cohorts. Furthermore, we could not perform force measurements of cardiomyocytes, and therefore the association of myocardial elasticity with the changes observed on the protein level can only be hypothesized. As mentioned previously, only total titin phosphorylation at the N2B domain was assessed, while the effect of titin phosphorylation can be highly dependent on the specific phospho-sites involved. In this regard, the present study cannot identify the specific signalling pathways responsible for the observed modifications.

## 5 Conclusion

We Observed significant changes in titin isoform ratio, total N2B-phosphorylation, oxidation and ubiquitination in patients with MVP and severe MR compared to healthy controls. Interestingly, disproportionate LV remodelling was showed a trend towards increased N2BA titin expression and total N2B-titin phosphorylation, both markers of decreased myocardial stiffness, compared to patients with normal LV remodelling. This study improves the pathophysiological insights underlying the process of LV remodelling in primary MR, which will improve our understanding of the disease and create opportunities for research regarding novel therapeutic targets in the future.

## Data Availability

The raw data supporting the conclusions of this article will be made available by the authors, without undue reservation.
