# Supplementary material for: Titin modulation and left ventricular remodelling in chronic primary mitral regurgitation
Source: Front Cardiovasc Med. 2026 Jun 3;13:1759286. doi: 10.3389/fcvm.2026.1759286 (PMC13272440; doi:10.3389/fcvm.2026.1759286)
Supplement: Supplementary file 1 [file DataSheet1.docx]

Supplementary Material

# Supplementary Figures


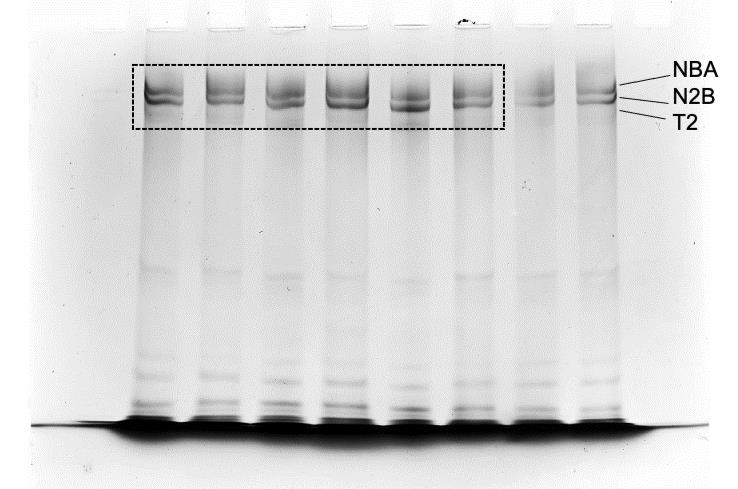


**Supplementary Figure 1:** Titin N2BA/N2B isoform ratio
Uncropped gel electrophoresis showing N2BA, N2B and T2 titin isoform bands.


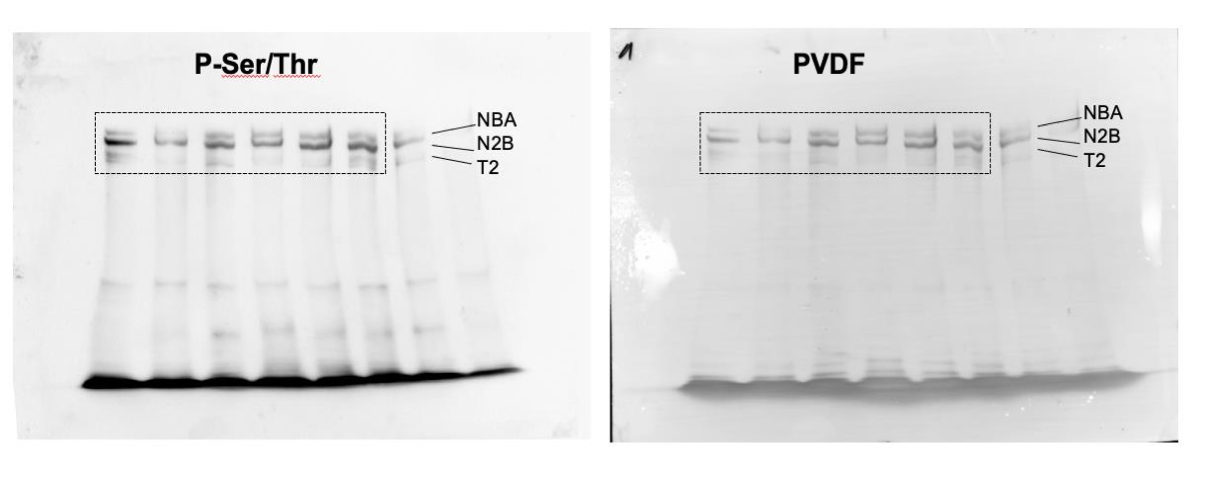
**Supplementary Figure 2:** Total N2B-titin phosphorylation
Uncropped immunoblot using anti–phospho‐serine/threonine antibodies (ECM Biosciences LLC; PP2551; 1:500) and PVDF stain as control.

A.
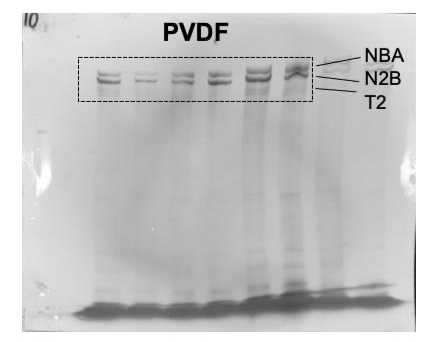

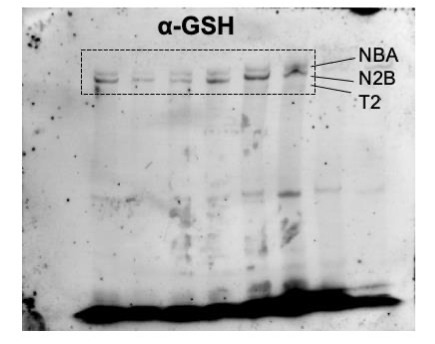


B.
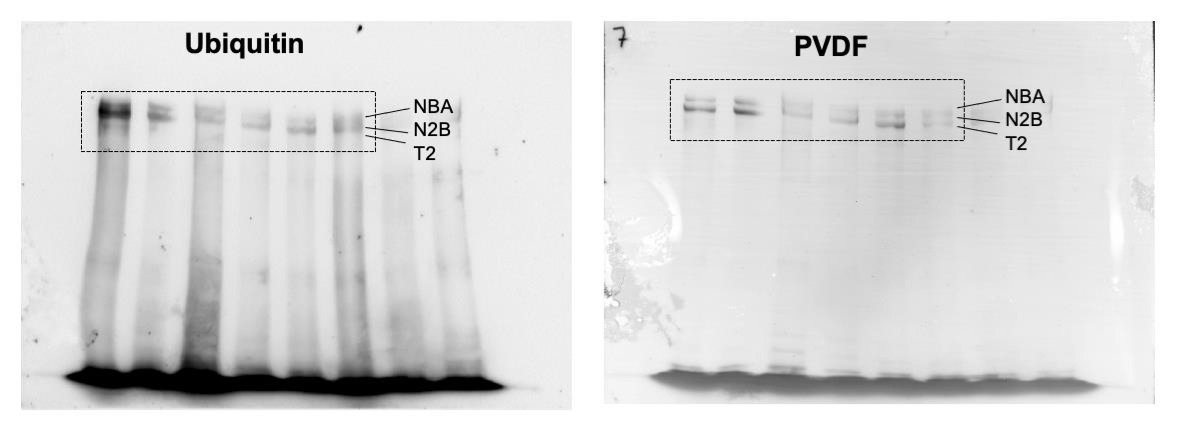


**Supplementary Figure 3:** Total N2B-titin glutathionylation and ubiquitination

Panel A: uncropped immunoblot using anti-GSH antibodies (ab19534, Abcam, 1:500) and PVDF stain as control.

Panel B: uncropped immunoblot using anti-ubiquitin antibodies (43124, Cell Signaling Technology, 1:1000) and PVDF stain as control.

# Supplementary Tables

Table S1: correlations between titin isoform ratio, phosphorylation, glutathionylation and ubiquitination with LV global longitudinal strain and myocardial fibrosis

|  | Ratio N2BA/N2B | | Total N2B-titin phosphorylation [a.u.] | | Total N2B-titin glutathionylation [a.u.] | | Total N2B-titin ubiquitination [a.u.] | |
| --- | --- | --- | --- | --- | --- | --- | --- | --- |
|  | *Correlation coefficient* | *P value* | *Correlation coefficient* | *P value* | *Correlation coefficient* | *P value* | *Correlation coefficient* | *P value* |
| LV GLS (%) | 0.259 | 0.416 | 0.361 | 0.249 | -0.438 | 0.155 | 0.357 | 0.254 |
| LGE present | -0.364 | 0.166 | -0.140 | 0.605 | -0.168 | 0.534 | -0.616 | **0.011** |
| ECV (%) | 0.359 | 0.208 | -0.046 | 0.875 | 0.125 | 0.669 | 0.246 | 0.396 |
| MVP subtype (=BD) | 0.392 | 0.133 | 0.168 | 0.534 | -0.028 | 0.918 | -0.448 | 0.082 |

ECV, extracellular volume; FED, fibroelastic deficiency; GLS, global longitudinal strain; LV, left ventricular; LGE, late gadolinium enhancement; MVP, mitral valve prolapse

Correlations are all performed using Spearman correlation (non-parametric).
